# Brothers in Affliction

**Published:** 1895-05-04

**Authors:** 


### Modern Sociology.

### BROTHERS IN AFFLICTION.

While much has been done for those afflicted with any definite ailment of mind or body, much remains to be done for those whose ailments lie between. Yet these are among the most distressing that afflict humanity, and are the more grievous the nearer the sufferer approaches to the normal type. The epileptic who is vigorous in mind and body between his attacks, the feeble-minded man or woman who just vaguely feels that he is different from his fellows, without being able to say wherein the difference lies, are more to be pitied than the madman who can soothe his brain with flattering fancies of his own wisdom and greatness, and less is done for them. We have just one hospital for the epileptic, and many want not a hospital but an asylum, or rather such an institution between the two as they may cheerfully take as their lifelong home, where they can work when their strength permits, and be nursed when they break down. For the feeble-minded, something more is wanted—a school, or rather a kindergarten where such faculties as they have may be developed and cultivated. But they have one thing in common. For neither is the home the best place.

In the home circle, every member has his or her own work, none can give the invalid the undivided attention which he needs. Nay, more, they cannot for ignorance of what is most desirable, and also from the mental repulsion which such affliction raises in the breasts even of kinsfolk. The epileptic is alarming in his fits; the feeble-minded is constantly irritating. Many a mother who would show herself tender to a child dying of consumption, or one whose mind was wholly gone, finds her patience tried past endurance by one who can almost do anything and yet succeeds in nothing. The feeble-minded, indeed, are not fit for conflict with the world. The feeble-minded girl is more easily seduced, the feeble-minded youth more easily cheated, or even led into crime, than another; they need, for a lifetime instead of a few years, the ever-watchful control, the educational discipline, the unwearying kindness of the nursery.

The first essential in the treatment of both these forms of brain disease is fresh air. Some centuries ago a number of Spanish monks provided an asylum for imbeciles. They reported that they could cure everyone but noblemen, because the rigorous etiquette of the nation forbade that these should be set to till the soil. The royal race of Spain is a pattern example of progressive mental disease, due, in some measure, perhaps, to this very etiquette that restrained the natural energies of both soul and body, and it may be that Charles V., the one great monarch of the race, saved himself from madness when he abdicated the throne and retired to plant cabbages in a monastery garden. A healthy country life is at least part of the cause of the success of the epileptic colony at Bielefield, of which we have a copy in England in the Maghull home, founded through the energy of Dr. William Alexander and the beneficence of Mr. Cox, of Liverpool. Other and newer institutions are that founded by the Countess of Meath at Godalming, and the Farm Colony recently established at Chalfont St. Peter. It has been suggested by an American philanthropist that these colonies might include both epileptics and feeble-minded. He evidently thinks that as both suffer from brain disease and neither is wholly incapable, they may conveniently be classed together. To a certain very limited extent this may be. An exceptional epileptic may, between his attacks, be entrusted with the care of his feeble-minded brethren. An exceptionally bright and sympathetic imbecile—we use the word for want of an accurate substantive by which to denominate those who just fail to reach the average standard of intellect—may be trusted to be the nurse and companion of an epileptic. But in most cases the very similarity of the causes of their maladies makes such association impossible. It is only too easy to imagine cases where the sight of an epileptic in a sudden fit might so frighten his imbecile companion and paralyse his senses as to bring about a fatal result to both.

The epileptic is a man foredoomed. His lot may be alleviated, his energies usefully employed between his attacks, but one day or another the access will arrive, and his condition will always be uncertain from hour to hour. The feeble-minded may, on the other hand, be raised by degrees to a permanent point of intelligence and usefulness. He may be made self-supporting—in some cases he may even become profitable to the community that maintains him, though this must not be looked for in every case. But what he needs most is not physical so much as moral guardianship, with education, patient, persistent and long-suffering, suited to his wants.

But certainly our American friend was right in holding up as an ideal in the education of the feebleminded the thought of helping others. Child-like, he loves the thought of helping, of being useful. Very touching is the story told by Mrs. Alice Mott of the effect on a low-grade idiot, of watching the growth of an ear of corn. He kept it as a treasure, hidden in a secret nook, and showed it only to his chosen friends. To these he would exclaim, when he displayed it, "with such tears as may have dimmed the eyes of Michael Angelo, 'I raised that!'" Conscious, though vaguely, of their separation from the rest of humanity, imbeciles find their highest joy in sharing its labours. This makes technical training of the first importance in the education of the feeble-minded. The sooner it is commenced, the better are the chances of the patient approaching the normal standard of humanity. You may make a boy into a fairly successful cobbler or carpenter, a girl into a seamstress or a laundress, whose work will hold its own with that of their happier fellows, if only they themselves are relieved from the cares and responsibilities of the world. But the process will be slow, and it will be wise not to expect from the imbecile of thirty more than you could demand of the normal youth of fifteen. And everything must be done under a closer, a kindlier, and a more intelligent supervision than that given to the average worker. Yet, even as a matter of national economy, it is desirable to draw these weaker brethren into institutions. If they can be made self-supporting, so much the better; but even if not, it is something if they are kept in happy celibacy, and prevented from reproducing a race which, in the end, will be a burden if not a danger to society.

